# Expanding the eligibility criteria for drugs in Canada’s time-limited health technology assessment and temporary drug access processes will further accelerate access to new medicines

**DOI:** 10.3389/jpps.2024.13694

**Published:** 2024-10-08

**Authors:** Allison Wills

**Affiliations:** 20Sense Corp., Toronto, ON, Canada

**Keywords:** health technology assessment, market access, reimbursement, patients, novel medications

## Background

Canada’s Drug Agency (CDA-AMC) conducts health technology assessments (HTA) for Canada’s federal, provincial, and territorial public drug programs (except Quebec’s) to guide their drug reimbursement decisions [1, 2]. The pan-Canadian Pharmaceutical Alliance (pCPA) conducts joint negotiations for Canada’s public drug plans [3]. It takes more than 1.5 years after regulatory approval for a new drug to be listed on the public plans in Canada – one of the slowest timelines among OECD20 countries [4, 5], many of which have established novel reimbursement decision pathways that enable publicly funded early access [6, 7]. Until recently, the CDA-AMC and pCPA processes did not include a pathway to help balance timely patient access with decision-making for drugs with high levels of uncertainty but promising early data [6, 7].

## New Canadian time-limited HTA and temporary drug access processes

In September 2023, the CDA-AMC announced a new time-limited drug reimbursement recommendation category that aims to “help provide earlier access to promising new treatments that target the unmet needs of people in Canada living with severe, rare, or debilitating illnesses.” [8] [CIT] In parallel, the pCPA developed a set of principles and conditions for a Temporary Access Process (pTAP) to “inform the negotiation process and potential product listing agreements for any drug products that follow” the CDA-AMC’s time-limited recommendation (TLR) pathway [9]. Together, these processes represent a significant development in addressing timely patient access as they form the first Canadian early access pathway.

## Current eligibility for TLR and pTAP

The drug eligibility criteria for TLR include a Notice of Compliance with Conditions (NOC/c) from Health Canada, plans to generate evidence for a phase III clinical trial in the same patient population as the original submission, and study completion within 3 years [8]. The same criteria apply to pTAP [9].

The CDA-AMC noted that the initial TLR eligibility is a “first step,” and plans to evaluate and refine the criteria based on experiences using the processes “after the first 3 to 5 recommendations have been issued or after 18 months, whichever is soonest.” [CIT] [8] pTAP is a “pilot project and will be subject to regular monitoring and assessment.” [9].

## First use of TLR and pTAP significantly reduces the time to listing

To date, one drug has leveraged these new processes [10]. On 28 November 2023, the CDA-AMC accepted the drug epcoritamab, for relapsed or refractory diffuse large B-cell lymphoma, as eligible for consideration of a TLR, and granted it a TLR on 18 June 2024 [11]. Negotiations between the manufacturer and the pCPA were concluded with a signed letter of intent on 19 July 2024 [12, 13]. First provincial listings were achieved on 14 August 2024, in Quebec and Ontario [14, 15] – 306 days after epcoritamab’s regulatory approval, a significantly earlier time-to-listing than the historical average, which is closer to 600 days for oncology therapies [4].

## Early observations: current eligibility criteria may limit pathway uptake

Almost 12 months after the launch of TLR and pTAP, it is encouraging to see that the first drug has successfully navigated these new processes, leading to timelier patient access. At the same time, this is the only drug to have leveraged this pathway to date – and it is uncertain whether other drugs will follow suit anytime soon. Of the estimated 2 NOC/c drug files that will receive approval by the end of 2024, one is currently going through the HTA process without leveraging TLR and the other file is pending at the time of writing [16–19].

Given the CDA-AMC’s goal of using TLR to expedite access for patients to promising new treatments, the current eligibility criteria may be too restrictive. If few drugs meet the eligibility criteria and even fewer apply, the TLR and pTAP processes will have a minimal impact.

## Considerations for the future evolution of TLR and pTAP drug eligibility criteria

Fortunately, there are many jurisdictions to learn from when considering adaptations of eligibility criteria for drugs that could increase TLR and pTAP use and impact. Examples abound in established early access pathways in OCED countries – for example, those at NICE, AIFA and HAS [6]; in emerging pathways, such as Taiwan’s conditional listing policy introduced in July 2023 [20] and, closer to home, in Quebec’s HTA agency INESSS’s “promise of value” recommendation option, which has been in place since 2018 and facilitates conditional listings that may include requirements for clinical monitoring and real-world data generation to support a subsequent re-evaluation [21]. Specifically for TLR and pTAP, stakeholders may consider the following:

1. NOC/c expansion to allow for other drug files. As previously noted, there will only be an estimated total of two NOC/c drug files approved by the end of 2024 [17]. Furthermore, a recent CDA-AMC analysis found that from 2018 to 2021, 94% of NOC/c authorisations issued were for oncology drugs [22]. Expanding eligibility beyond drugs that receive NOC/c would allow for a greater number of therapies that meet a high unmet patient need – including those for rare diseases and conditions – to leverage this pathway.
2. Incorporating greater flexibility into the Phase III clinical trial requirements by allowing the use of phase III data where the patient population, line of therapy, and/or indication do not fully align with the phase II trial data provided in the original HTA submission. This change alone could make up to 50% more files eligible for TLR [23].
3. Accepting real-world evidence (RWE) as a supplement to clinical trial data. As recent examples of RWE use in a timely access process in Canada, in the May 2024 INESSS reassessments of two therapies with initial recommendations based on the promise of value, the confirmatory data included both clinical trial data and eight real-world studies – three of which included RWE from Canadian patients [24, 25].

## Encouraging progress – but is it enough to meaningfully improve timely access for patients?

The current TLR and pTAP processes represent an encouraging step forward towards modernising Canada’s approach to HTA and drug reimbursement – a priority noted recently by Ontario premier Doug Ford [26].

It is hoped that TLR and pTAP will continue to evolve through the integration of learnings from early experiences, the study of other jurisdictions’ early access pathways, and input from and transparent collaboration of Canadian stakeholders – key to achieving a “made-in-Canada solution” [27] that supports evidence-based decision making and timely patient access to promising medicines. To ensure that TLR and pTAP can fulfil their potential and reach the greatest number of patients in need, the expansion of drug eligibility criteria would be a logical next step.

## Abbreviations

AIFA, Agenzia Italiana del Farmaco; CDA-AMC, Canada’s Drug Agency – L’agence des médicaments du Canada; HAS, Haute Autorité de Santé; HTA, Health technology assessment; INESSS, L’Institut national d’excellence en santé et en services sociaux; NOC/C, Notice of Compliance with Conditions; NICE, National Institute for Health Care and Excellence; OECD, Organisation for Economic Co-operation and Development; pCPA, pan-Canadian Pharmaceutical Alliance; pTAP, pan-Canadian Pharmaceutical Alliance Temporary Access Process; RWE, Real-world evidence; TLR, Time-limited recommendation.
